# Mating-induced Ecdysone in the testis disrupts soma-germline contacts and stem cell cytokinesis

**DOI:** 10.1242/dev.202542

**Published:** 2024-06-04

**Authors:** Tiffany V. Roach, Kari F. Lenhart

**Affiliations:** Department of Biology, Drexel University, Chestnut St, Philadelphia, PA 19104, USA

**Keywords:** Mating, Cytokinesis, Stem cells, Ecdysone, Encystment, *Drosophila*

## Abstract

Germline maintenance relies on adult stem cells to continually replenish lost gametes over a lifetime and respond to external cues altering the demands on the tissue. Mating worsens germline homeostasis over time, yet a negative impact on stem cell behavior has not been explored. Using extended live imaging of the *Drosophila* testis stem cell niche, we find that short periods of mating in young males disrupts cytokinesis in germline stem cells (GSCs). This defect leads to failure of abscission, preventing release of differentiating cells from the niche. We find that GSC abscission failure is caused by increased Ecdysone hormone signaling induced upon mating, which leads to disrupted somatic encystment of the germline. Abscission failure is rescued by isolating males from females, but recurs with resumption of mating. Importantly, reiterative mating also leads to increased GSC loss, requiring increased restoration of stem cells via symmetric renewal and de-differentiation. Together, these results suggest a model whereby acute mating results in hormonal changes that negatively impact GSC cytokinesis but preserves the stem cell population.

## Teaser

**Highlighted Article:** Mating increases Ecdysone signaling in soma of the testes that disrupts soma-germline contacts and stem cell cytokinesis but aids in retention of the stem cell pool over time.

## INTRODUCTION

Tissue homeostasis is accomplished by balancing stem cell self-renewal and production of differentiating daughter cells. Tissues engage multiple mechanisms to uphold this balance under stressful conditions, including de-differentiation and increased stem cell cycling (Tower, 2012). Mating is a natural physiological process but can be detrimental to tissue homeostasis (Maklakov and Immler, 2016). Increased production of sperm or eggs depends on the ability of stem cells to respond to and compensate for demand. Long-term mating combined with aging or nutrient deprivation results in loss of stem cells or overproliferation of undifferentiated cells (Chang et al., 2019; Herrera and Bach, 2021). However, much less is known about the proximate effects of mating stress, alone, on stem cell biology. Although some changes in stem cell behaviors can be identified from fixed tissue (Ameku and Niwa, 2016; Malpe et al., 2020), these analyses are limited in the ability to resolve acute changes in cellular dynamics.

**Fig. 1.**
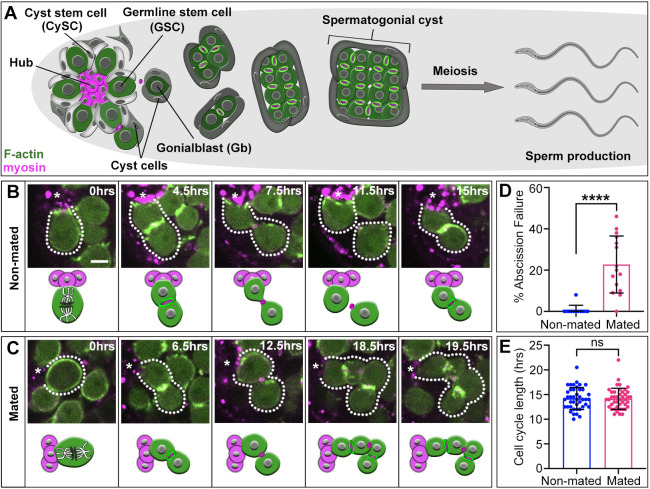
**Mating causes a significant increase in GSC abscission failure.** (A) Diagram of testis niche and germline development. (B,C) Time-lapse of nanos-ABD-moe::GFP (green) and Myo-mCherry (magenta) during GSC divisions in non-mated (B) and mated (C) animals. Each image is 1-4 *z*-slices. Asterisks indicate niche. Dotted lines indicate a GSC and daughter(s) as they go through two rounds of mitotic division. (D) Percent GSC abscission failure under non-mating (*n*=12 testes; failed abscission: 1/135 divisions) and mating conditions (*n*=15 testes; failed abscission: 40/167 divisions). (E) GSC cell cycle length in non-mated (*n*=42 GSCs in 10 testes) and mated animals (*n*=43 GSCs in 14 testes). ****P<0.0001 (non-parametric Mann–Whitney *U*-test). ns, not significant. Error bars: s.d. All experiments *n*≥2 trials. Scale bar: 5 µm (for B,C).

The *Drosophila* testis niche is an ideal model to address how physiological changes directly impact stem cells. The testis contains two stem cell populations: germline stem cells (GSCs) and somatic cyst stem cells (CySCs). As in most animals, there is an obligatory association between soma and germline (Lui et al., 2003). In the fly testis, germ cell differentiation requires full encapsulation of each GSC daughter by two somatic cyst cells of the CySC lineage in a process called encystment (Fig. 1A) (Sarkar et al., 2007; Kiger et al., 2000). Soma-germline contacts are then stabilized and maintained by adherens and septate junctions as they co-differentiate (Sahu et al., 2021; Fairchild et al., 2016). Using extended live imaging of the stem cell niche, we find that mating disrupts GSC cytokinesis by decreasing soma-germline contacts. Abscission failure caused by defective encystment is mediated by increased Ecdysone signaling upon mating, is significantly reduced when mating stress is removed, and recurs when mating is reintroduced, demonstrating a consequential impact on germline homeostasis.

## RESULTS AND DISCUSSION

### Mating disrupts release of GSC daughters from the niche

Mating increases GSC numbers and cycling rates, but these changes do not explain how mating contributes to tissue degeneration or tumorigenesis (Ameku and Niwa, 2016; Malpe et al., 2020; Chang et al., 2019). Aging stress causes early defects in GSC cytokinesis, leading to reduced numbers of differentiating germ cells – a hallmark of diminished homeostasis (Lenhart et al., 2019). To address whether mating impacts GSC cytokinesis, we performed live imaging to visualize GSC divisions and cytokinesis in testes of males that underwent 2 days of mating in a 3:1 virgin female-to-male ratio. In non-mated males, GSCs execute a reproducible but modified cytokinetic program (Lenhart and DiNardo, 2015). Following mitosis, the actomyosin contractile ring is replaced by a secondary F-actin ring at the intercellular bridge between the GSC and daughter (Fig. 1B), which prevents cytokinesis progression until its disassembly. At this point, cytokinesis resumes and completes with the final severing of membranes between daughter cells (abscission). Under homeostatic conditions GSC abscission is robust, completing in ∼100% of cells (Fig. 1D). By contrast, despite progressing normally through early cytokinesis, GSCs from mated males frequently fail to complete abscission (Fig. 1C,D, *P*<0.0001). This results in a chain of connected daughter cells remaining attached to the niche (Fig. 1C), preventing release of differentiating germ cells. Thus, despite increased need for germ cells upon mating, our data suggest the immediate effect on GSCs decreases the release of daughters from the niche.

Previous studies analyzed mitotic or synthesis (S) phase indices on fixed tissues and found that even short-term mating can induce a faster cycling rate in male and female GSCs (Ameku and Niwa, 2016; Malpe et al., 2020). As we previously showed that fast cycling causes abscission failure (Lenhart et al., 2019), we asked whether the mating-induced cytokinetic defects in GSCs result from altered cycling rates. Our 24-h imaging allowed us to directly quantify cell cycle lengths of individual GSCs. Consistent with existing literature, we observed variability in cycling length (10-22 h) with an average rate of 14 h (Fig. 1E) (Gadre et al., 2021; Lenhart and DiNardo, 2015; Lenhart et al., 2019). We found that GSCs from mated males had both similar variability and identical average cycling rates compared with non-mated males (Fig. 1E, *P*=0.8418). To confirm these results, we determined the S phase index of GSCs in fixed tissue by exposing testes to 5-ethynyl-20-deoxyuridine (EdU) before fixation. Again, we found no significant difference in GSC S phase index between non-mated and mated testes (Fig. S1, *P*=0.9102). Together, these data suggest that mating-induced abscission failure is not caused by increased cycling rates in GSCs.

### Mating causes defects in somatic encystment of germ cells

**Fig. 2.**
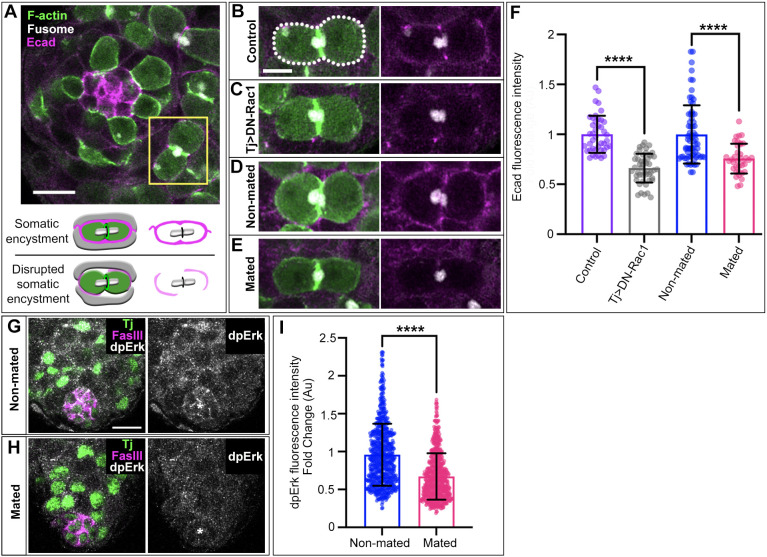
**Mating leads to decreased soma-germline adhesion and EGFR signaling.** (A) Apical tip of the testis showing two-cell cyst (yellow box) and diagram of normal versus disrupted somatic encystment. (B-E) Magnified view of two-cell cysts from a control and non-mated testis showing typical E-cad accumulation at soma-germline junctions (white dotted line) (B,D) and a testis with somatic expression of DN-Rac1 and mated testis showing diminished E-cad levels at soma-germline contacts (C,E). (F) Quantification of E-cad fluorescence intensity represented as fold change relative to the respective controls (*n*≥40 two-cell cysts, minimum of 10 testes). (G,H) Immunofluorescent staining of somatic nuclei (green, Tj), niche cell junctions (magenta, FasciclinIII/FasIII) and dpErk (gray) in a non-mated testis (G) and a mated testis with diminished dpErk (H). (I) Quantification of dpErk fluorescence intensity in somatic nuclei represented as fold change (*n*≥345 somatic nuclei in at least six testes). ****P<0.0001 (non-parametric Mann–Whitney *U*-test). Error bars: s.d. All experiments *n*≥2 trials. Scale bars: 10 µm (A,G,H); 5 µm (B-E).

We previously identified a crucial role for somatic encystment in the abscission step of GSC cytokinesis (Lenhart and DiNardo, 2015). Thus, we investigated whether soma-germline interactions are disrupted upon mating. Current analyses of encystment rely on visualizing large morphological changes or full-tissue quantification of adhesion proteins. However, our previous work indicates that testes with subtle encystment disruptions can appear to be wild type in morphology while having significant effects on the stem cell population. Specifically, disrupting the actin cytoskeleton in somatic cells through expression of a dominant negative Rac1 (DN-Rac1) does not disrupt overall organization of the soma and germline but is sufficient to cause significant GSC abscission failure (Lenhart and DiNardo, 2015). Therefore, we established a method for detecting subtle disruptions in soma-germline contacts focusing on two-cell cysts, as encystment is complete by this stage (Fig. 2A). We quantified the mean fluorescence intensity of the adherens junction protein E-cadherin (E-cad) specifically at soma-germline interfaces and determined the fold change in fluorescence relative to controls (Fig. 2B-E). First, we validated this method in testes with soma expression of DN-Rac1 (Fig. 2C). Consistent with our previous data suggesting that this manipulation subtly disrupts encystment (Lenhart and DiNardo, 2015), we observed significantly reduced E-cad intensities at soma-germline junctions compared with controls (Fig. 2B,C,F). Interestingly, quantification of E-cad in non-mated versus mated males also revealed a significant decrease in adherens junction formation between soma and germline (Fig. 2D-F, *P*<0.0001). Importantly, we quantified the intensity of E-cad at GSC-niche interfaces and found no difference between mated and non-mated males (Fig. S2). Together, these data suggest a specific decrease in soma-germline contacts due to mating.

Encystment is mediated by epidermal growth factor receptor (EGFR) activation on somatic cell membranes, which triggers two intracellular pathways: (1) activation of Rac1, which enables actin rearrangements to encapsulate germ cells; (2) transcriptional changes through phosphorylation of Erk/MAPK (Sarkar et al., 2007; Kiger et al., 2000). The fluorescence intensity of dpErk immunostaining is commonly used as a readout for the level of EGFR activation impacting encystment (Fairchild et al., 2016). We found a significant decrease in somatic nuclear dpErk in mated compared with non-mated males (Fig. 2G-I), indicating decreased EGFR activity. Together, these data strongly suggest that mating disrupts somatic encystment of the germline, causing GSC abscission failure.

### Germ cell depletion does not cause GSC abscission failure

**Fig. 3.**
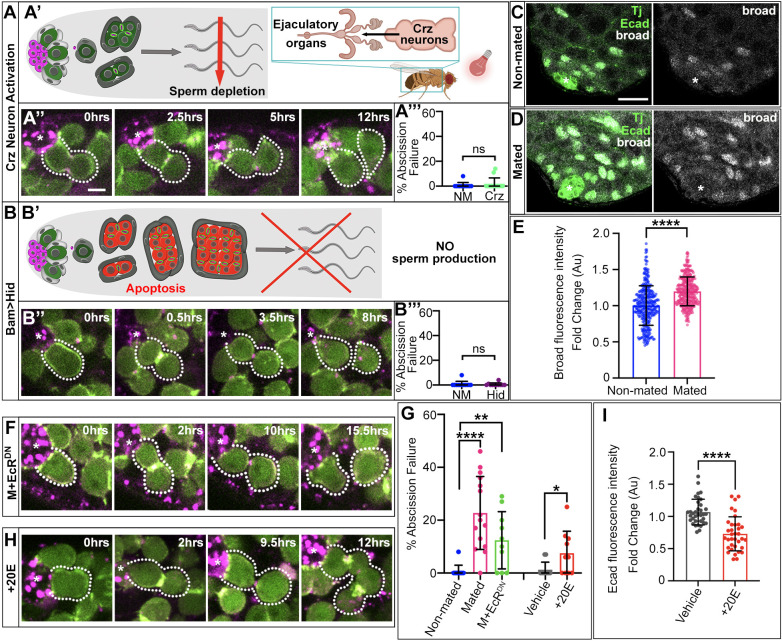
**Ecdysone signaling but not germ cell depletion causes GSC abscission failure.** (A,B,F-H) Time-lapse of nanos-ABD-moe::GFP (green) and Myo-mCherry (magenta) during GSC divisions in testes of males with optogenetic activation of Crz neurons (A″), expressing Bam-Hid (B″), fed 20E (F) and mated with somatic expression of DN-EcRB2 (H). Dotted lines indicate a GSC and daughter as they progress through successful (A″,B″,F) or failed (H) cytokinesis. (A′,B′) Diagrams of methods for sperm/germ cell depletion in the absence of mating. (A‴,B‴,G) Quantification of GSC abscission failure (*n*≥11 testes) (non-mated/mated data repeated from Fig. 1). (C,D) Immunostaining of Tj, E-cad and Broad in non-mated and mated males. (E) Quantification of broad fluorescence intensity represented as fold change (*n*≥345 somatic nuclei in at least six testes). (I) Quantification of E-cad fluorescence intensity at soma-germline contacts of two-cell cysts represented as fold change relative to the vehicle fed controls (*n*=35 two-cell cysts in 10 testes). **P<0.01, ****P<0.0001 (non-parametric Mann–Whitney *U*-test). ns, not significant. Error bars: s.d. All experiments *n*≥2 trials. Scale bars: 5 µm (A″,B″,F,H); 10 µm (C,D).

Previous work has shown that changes in GSC biology with mating are induced by the act of mating itself, not by pheromone sensing or exposure to courtship behavior (Malpe et al., 2020). However, which physiological effect of mating is responsible for altered stem cell behavior remains unclear. At the tissue level, the most crucial consequence of mating is depletion of the differentiating germ cell population, which could feed back to change GSC behavior. Therefore, we tested whether depletion of sperm in the absence of mating alters GSC cytokinesis. We triggered ejaculation by optogenetically activating Crz neurons expressing red-shifted channelrhodopsin CsChrimson, thereby forcing depletion of sperm in the absence of mating (Fig. 3A′) (Zer-Krispil et al., 2018; Tayler et al., 2012). We confirmed that this protocol results in a similar reduction in sperm density within seminal vesicles to that observed in mated males (Fig. S3A-C). However, we did not observe significant GSC abscission failure upon optogenetic triggering of ejaculation (Fig. 3A″,A‴). We next tested whether a more severe depletion of differentiated germ cells was sufficient to induce GSC abscission failure in the absence of mating. By expressing an apoptotic factor, Hid, in differentiating germ cells via Bam-Gal4, we can retain the niche and resident stem cells, but effectively kill all germ cells past the two-cell cyst stage (Fig. 3B′; Fig. S3D,F). As a result, Bam>Hid testes lack sperm and are significantly smaller than control testes (Fig. S3E,G). Surprisingly, we found that catastrophic loss of differentiating germ cells did not cause GSC abscission failure (Fig. 3B″,B‴). Together, these data suggest that depletion of germ cells does not induce abscission failure and that a different aspect of mating causes defects in the stem cell pool.

### Mating increases Ecdysone signaling, which is necessary and sufficient to cause GSC abscission failure

Ecdysone signaling increases in the female gonad following mating, causing faster GSC divisions (Ameku and Niwa, 2016). Although male accessory organs have increased biosynthesis of Ecdysone during mating (Pondeville et al., 2008), whether released Ecdysone affects the testis or male GSC function is unknown. Ecdysone and EGF signaling have been shown to act antagonistically in male somatic cells, with Ecdysone negatively impacting EGFR activity (Qian et al., 2014). Thus, we investigated whether mating increases Ecdysone signaling in testis soma.

To assay Ecdysone activity, we quantified fluorescence intensity of the known Ecdysone target, Broad-core (Broad) (Li et al., 2014). We identified somatic nuclei by staining for Traffic jam (Tj) and quantified Broad levels between non-mated and mated testes (Fig. 3C,D). Mating induced a significant increase in Broad accumulation (Fig. 3E), suggesting that Ecdysone signaling in soma is indeed elevated. To determine whether this increased Ecdysone is necessary for mating-induced abscission failure, we expressed a dominant negative EcR-B2 in testis soma under mating conditions. Excitingly, a decrease in EcR-B2 activity significantly rescued GSC abscission failure with mating (Fig. 3F,G).

To address whether increased Ecdysone is sufficient to induce abscission failure, we fed males the steroid hormone 20-hydroxyecdysone (20E) to increase signaling in the absence of mating (Li et al., 2014; Domanitskaya et al., 2014). Flies were fed a mixture of apple juice and blue dye supplemented with either vehicle control (1% ethanol, lacking 20E) or 20E on filter paper each day for 2 days to closely mimic the mating-induced increases in Ecdysone signaling, confirmed by increased Broad expression (Fig. S3H). We found that 20E feeding in the absence of mating was sufficient to significantly increase the incidence of GSC abscission failure (Fig. 3G,H). Quantification of E-cad also revealed significantly decreased levels at soma-germline contacts in testes of 20E-fed males (Fig. 3I). Taken together, our data suggest that increased Ecdysone signaling is the proximate cause of encystment disruptions and GSC abscission failure upon mating.

### Mating-induced GSC abscission failure is transient

**Fig. 4.**
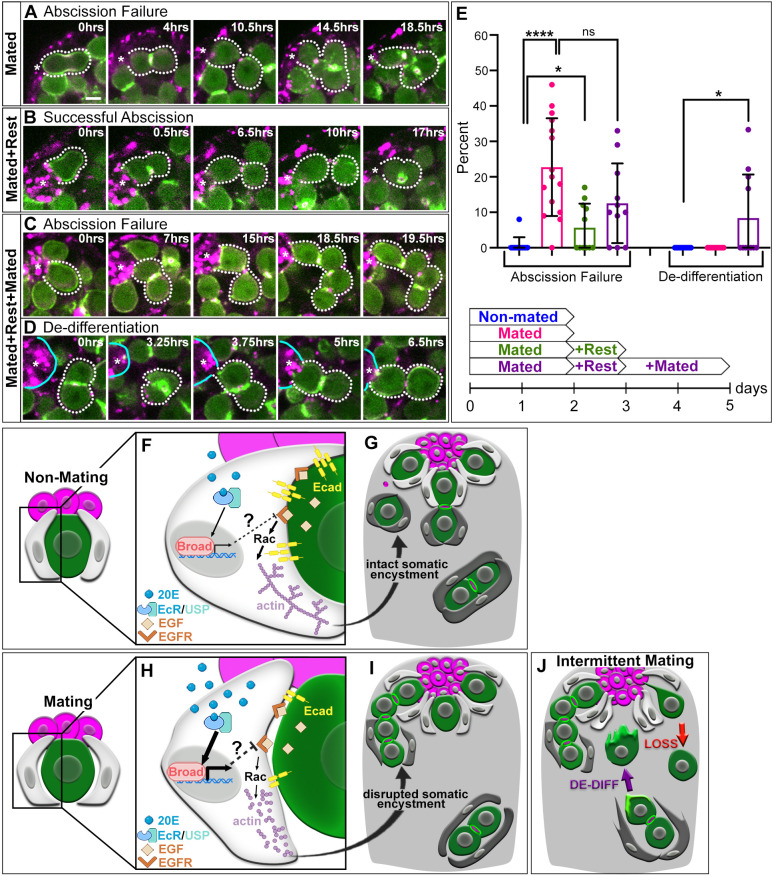
**Mating-induced GSC abscission failure is transient but reoccurs with repeated bouts of mating.** (A-C) Time-lapse of nanos-ABD-moe::GFP (green) and Myo-mCherry (magenta) during GSC divisions in testes of 2 days mated (A) 2 days mated+1 day of rest (B) and 2 days mated+1 day of rest+2 days mated (C) males. (D) De-differentiation of a two-cell cyst in 2 days mated+1 day of rest+2 days mated testes. Dotted lines indicate a GSC and daughter as they undergo failed (A,C) or successful (B) abscission, and a two-cell cyst that de-differentiates and reenters the niche (D). (E) Quantification of GSC abscission failure and de-differentiation (*n*≥11 testes). (F-I) Model for Ecdysone-mediated control of somatic encystment in non-mating (F,G) and mating (H,I) conditions. (J) Model of the potential adaptive function for decreased encystment. *P<0.05, ****P<0.0001 (non-parametric Mann–Whitney *U*-test). ns, not significant. Error bars: s.d. All experiments *n*≥2 trials. Scale bar: 5 µm.

In female flies, the effect of Ecdysone on GSCs upon mating is acute and resolves as soon as sperm are lost from the seminal receptacle (Ameku and Niwa, 2016). To determine whether the effects of mating on male GSCs are also transient, we allowed flies to rest for 1 day in the absence of females after extensive mating and performed live imaging to assess GSC cytokinesis. Although not completely restored to non-mated levels, we found that GSC abscission failure was significantly reduced after only 1 day of rest (Fig. 4A,B,E), suggesting that these defects are also transient and resolve when the stress of mating is removed.

### Mimicking natural mating events causes abscission failure and de-differentiation to restore the stem cell pool

Intermittent bouts of mating are likely the natural experience of flies in the wild. Previous work has shown that simulating natural mating over an extended period induces GSC loss and de-differentiation of germ cells to replenish the stem cell pool (Herrera and Bach, 2021). To investigate the early effects of natural mating on the GSC population, we allowed males to mate for 2 days, followed by a day of rest, and then repeated 2 days of mating. Interestingly, when flies underwent a second round of extensive mating, the rate of abscission failure, again, significantly increases (Fig. 4C,E). Additionally, in this brief mating paradigm, we observed germ cell phenotypes normally seen with prolonged natural mating, including an increase in GSC loss and restoration of GSCs through an increase in frequency of symmetric renewal, where a newly produced GSC daughter rotates into the niche and becomes a stem cell (Fig. S4). Importantly, we also observed seven instances of de-differentiation – a behavior never observed in non-mated testes (Fig. 4D,E). Together, these data suggest that even short bouts of natural mating cause stem cell defects requiring induction of mechanisms to restore the stem cell pool.

Our data support a model in which extensive, short-term mating induces a burst of Ecdysone in males, just as in females. However, rather than increasing GSC number as in the ovary, increased Ecdysone signaling in the testis causes GSC abscission failure, resulting from decreased EGFR pathway activation. As EGFR signaling is the crucial pathway for Rac1-dependent cytoskeletal changes required for germline enclosure, lowered EGFR activity upon mating impairs somatic encystment, leading to decreased E-cad accumulation at soma-germline interfaces. Ultimately, defects in somatic encystment prevent robust completion of GSC abscission, leading to retention of GSC daughters at the niche and decreased release of differentiating germ cells (Fig. 4F-I).

We have identified an immediate, detrimental effect of mating on the GSC population of the testis that compromises homeostatic conditions. Despite mating increasing demand for sperm, this immediate defect decreases release of differentiating germ cells from the niche. What could explain this contradiction? One possibility is that enough sperm is still produced despite abscission failures to prevent selection against this acute defect in response to Ecdysone. However, a more intriguing possibility is that slight decreases in encystment upon mating might be beneficial to the tissue. Previous work has shown that de-differentiating germ cells become highly protrusive as they move back to the niche – something that cannot happen if those cells were still tightly encysted by soma (Sheng et al., 2009; Sheng and Matunis, 2011). Indeed, our own live imaging reveals protrusions in de-differentiating germ cells before contacting the niche (Fig. 4D), suggesting that decreases in somatic encystment, although negatively impacting GSC abscission, might serve an adaptive function to retain the stem cell pool by facilitating de-differentiation (Fig. 4J). It will be interesting in future to explore the necessity of diminished encystment for promoting germ cell de-differentiation. Importantly, persistent loss of encystment can cause overproliferation of germ cells (Shields et al., 2014). Therefore, what may promote plasticity early to preserve stem cells might also explain why mated males are susceptible to developing tumors with age (Chang et al., 2019). Lastly, our work provides new insights into the acute effects of mating stress on the testis and reveals a role for hormonal signaling, but not germ cell loss, on the regulation of soma-germline interactions. Given the requirement for soma-germline interactions during sperm production across species (Lui et al., 2003), it will be interesting to determine the degree of conservation in these effects of mating on stem cell biology and germline plasticity in other systems.

## MATERIALS AND METHODS

### Experimental model and subject detail

*Drosophila melanogaster* stocks were maintained on Bloomington *Drosophila* Stock Center (BDSC) standard cornmeal medium in vials or bottles. All crosses and males subjected to mating protocols were kept at 25°C unless otherwise indicated. Fly stocks included: Traffic Jam Gal4 (Kyoto Stock Center) to drive transgene expression in somatic cells of the testis; nos-ABDmoe::GFP (gift from Ruth Lehmann, Massachusetts Institute of Technology, USA) to visualize F-actin in early stage germ cells in live imaging experiments; *sqh*::mCherry^A11^ (gift from Adam Martin, Massachusetts Institute of Technology, USA) to visualize myosin localization in live imaging experiments; Crz Gal4 (gift from Lisa Shao, University of Toronto, Canada; BDSC: 51976; w[1118]; P{w[+mC]=Crz-GAL4.391}3M) to drive transgene expression in a subset of neurons responsible for stimulating ejaculation in the absence of mating; UAS CsChrimson (gift from Lisa Shao; BDSC: 55136; w[1118]; P{y[+t7.7] w[+mC]=20XUAS-IVS-CsChrimson.mVenus}attP2) to sensitize neurons for optogenetic stimulation with red LED. Stocks from the BDSC included: UAS DN-Rac1 (BDSC: 6292; y[1] w[*]; P{w[+mC]=UAS-Rac1.N17}1) to express in somatic cells of the testis and disrupt their encystment of germ cells; Bam Gal4 (BDSC: 80579; y[1] w[*] P{w[+mC]=bam-GAL4:VP16}1) to express an apoptotic transgene in early differentiating germ cells; UAS Hid (BDSC: 65403; P{w[+mC]=UAS-hid.Z}2/CyO) to express in early differentiating germ cells in the absence of mating; EcR-B2^DN^ (BDSC: 9450; w[*]; P{w[+mC]=UAS-EcR.B2.F645A}TP1) to decrease EcR activity in somatic cells of the testis; don juan::GFP (BDSC: 5417; w[*]; P{w[+mC]=dj-GFP.S}AS1/CyO) to label sperm.

### Mating protocol

For mating, 0- to 3-day-old males underwent 2 days of mating in a 3:1 virgin female to male ratio per vial (flipping onto new food and replacing female virgins with new ones each day) – which we refer to as extensive or acute mating. Age-matched non-mated virgin males were kept together in a vial in tandem and treated identically. Male courtship was confirmed to occur throughout the full 2 days of mating. At the end of the mating paradigm, all males were sacrificed, and their testes dissected for live imaging.

### Time lapse imaging

Extended time-lapse imaging and culture conditions were adapted from those previously described (Lenhart and DiNardo, 2015; Lenhart et al., 2019; Sheng and Matunis, 2011). Testes were dissected from 0- to 3-day-old males in Ringers solution and mounted onto a poly-lysine-coated coverslip at the bottom of an imaging dish (MatTek). Ringers solution was removed and imaging media (15% fetal bovine serum, 0.5× penicillin/streptomycin, 0.2 mg/ml insulin in Schneider's insect media) was added. Testes were imaged every 15 or 30 min for up to 24 h on an Olympus iX83 with a Yokagawa CSU-10 spinning disk scan head, 60×1.4 NA silicon oil immersion objective and Hamamatsu EM-CCD camera using 1 μm *z*-step size (40 μm stacks). Experiments were repeated a minimum of two times and at least ten testes were analyzed for each genotype/condition.

### Analysis of GSC and germ cell behaviors from live imaging

Abscission failure was defined as a GSC that entered mitosis while remaining connected to its daughter cell by a stable intercellular bridge for at least two consecutive time-points; whereas successful abscission was defined as an individual GSC that completely separated from its daughter cell before entering its next mitosis. The rate of abscission failure was calculated by dividing the total number GSC divisions with abscission failure by the total number of GSC divisions per testis. For cell cycle timing, we quantified M phase as visual production of two daughter cells (telophase or after) and the length of the cell cycle was determined for those GSCs in which we observed daughter cell production twice within the same imaging period. For quantification of either symmetric renewal or de-differentiation, the total number of GSCs acquired through each event was divided by the total number of GSCs at the end of the imaging session and reported as a percentage. For quantification of GSC loss, the total number of GSCs which lost contact with the hub were divided by the total number of GSCs at the beginning of the imaging and reported as a percentage.

### S phase labeling and analysis

To label cells in S phase, testes were dissected and incubated in 10 μM of EdU for 15 min before fixation. Testes were processed for immunohistochemistry (below), followed by visualization of the Edu label with Click-it Alexa Fluor 647 Edu kit (Invitrogen). An S phase index was determined by dividing the total number of GSCs that were Edu^+^ by the total number of GSCs per testis for each condition.

### Immunostaining

Immunostaining was performed as previously described (Lenhart and DiNardo, 2015; Lenhart et al., 2019; Terry et al., 2006). In short, testes were dissected in Ringers solution and fixed for 30 min in 4% formaldehyde in Buffer B (75 mM KCl; 25 mM NaCl; 3.3 mM MgCl_2_; 16.7 mM KPO_4_) followed by multiple washes in PBSTx (1× PBS, 0.1% Triton-X 100) and blocking in 2% normal donkey serum. Testes were incubated in primary antibodies at 4°C at least overnight, washed multiple times, and then incubated in appropriate secondary antibodies for 1 h at room temperature. After additional washes, testes were equilibrated in a solution of 50% glycerol and then mounted on slides in a solution of 80% glycerol. Primary antibodies used were: rat anti-DE-cadherin [Developmental Studies Hybridoma Bank (DSHB), 1:20], mouse anti-1B1 (DSHB, 1:50), chick anti-GFP (Aves Labs, 1020, 1:1000), guinea pig anti-traffic jam (Dorothea Godt, University of Toronto, Canada, 1:5000), mouse anti-fasciclin III (DSHB, 1:50), rabbit anti-phospho-p44/42 MAPK Erk (1/2) (Cell Signaling Technology, 9101, 1:100) and mouse anti-broad-core (25E9.D7)-s (DSHB, 1:50). Secondary antibodies used were from Jackson ImmunoResearch and used at 1:125 dilution: Alexa fluor-488 (anti-rabbit 711-545-152, anti-guinea pig 706-545-148, anti-rat 715-545-151), -Cy3 (anti-mouse 715-165-153) and -Cy5 (anti-rat 712-605-153, anti-guinea pig 706-175-148). All antibodies have been previously verified by the *Drosophila* community.

Immunolabeling against dpErk was performed using the methods described above with the following exceptions. Washes were carried out using PBS supplemented with phosphatase inhibitor cocktail 2 (Sigma-Aldrich, P5726, 1:100). This mixture was used in place of Buffer B for the 30 min fixation. Dissections were conducted on ice and each incubation/wash step was carried out at 4°C.

### Quantification of fluorescence intensities

For analysis of E-cad accumulation, we traced the soma-germline interfaces of two-cell cysts using the freehand line tool with a pixel width of 3 in ImageJ. Additionally, three niche-niche interfaces were measured and averaged as an internal reference as well as three background traces to subtract. Thus, the following formula was used: (mean two-cell cyst trace – mean average background) / (mean average reference – mean average background). The average mean for all two-cell cysts from the non-mated testes was determined and used to normalize each value from both non-mated and mated testes to reveal fold change differences.

For both dpErk and Broad analyses, regions of interest were drawn around somatic nuclei as defined by Tj accumulation and then mean fluorescence intensity of dpErk and Broad were measured separately in ImageJ. Again, the average mean for all Tj^+^ cells within 50 μm from the testis tip in the non-mated testes was determined and used to normalize each value from both non-mated and mated testes to reveal fold change differences. All experiments and respective controls were carried out in tandem and imaged with the same acquisition settings. For each genotype, a minimum of six testes and 345 nuclei were analyzed across at least two independent trials.

All images of fixed and immunostained testes were acquired using a Leica Stellaris 5 DMi8 inverted stand with tandem scanner; four power HyD spectral detectors; and HC PL APO 63×/1.4NA CS2 oil objective using LAS X software.

### Optogenetic activation

The parental cross (UAS CsChrimson×Crz Gal4) was reared on food supplemented with 1:500 all trans-Retinal in ethanol in the dark at 25°C. Newly eclosed virgin males carrying the UAS CsChrimson and Crz Gal4 were collected onto food containing 1:250 all trans-Retinal (R2500, Sigma-Aldrich) for 3 days. Then, while in the dark, males were anesthetized on ice, transferred to empty vials and allowed 5 min to sufficiently wake. Next, males were exposed to red LED light (∼650 nm, RedBeam™ Mini) for three 15 min activations with at least 45 min of rest in their respective food vials in between exposures (Zer-Krispil et al., 2018). We visually confirmed successful activation of neurons and resulting ejaculation by briefly exposing experimental and sibling control flies from each experiment to red light; consistently, 100% of experimental flies immediately exhibited behaviors consistent with ejaculation and this was observed in 0% of sibling controls. For experiments, the light was positioned to shine directly into the vial containing males and wrapped in foil to restrict ambient light. Additionally, this apparatus was placed in a black box at room temperature for the duration of the experiments. This procedure was repeated for 2 consecutive days to closely mimic the mating experience of our acute mating paradigm and males were subsequently sacrificed for live imaging. Using don juan::GFP, we confirmed that our optogenetic protocol induced similar depletion of sperm from seminal vesicles as the mating paradigm.

### Ecdysone feeding experiments

We fed 0- to 3-day-old virgin males a mixture of 20E (Sigma-Aldrich, H5142, 1 mM), 1% ethanol, 100% organic apple juice (Nature's Promise) and blue dye (McCormick) on filter paper. Half the males were fed the same mixture but lacking 20E. The food vials, filter papers and mixtures were replaced each day for 2 days to match the timeframe of the acute mating protocol. Following this, testes were dissected for live imaging.

### Quantification, statistical analysis and image processing

Time-lapse images were analyzed and *z*-projections generated using ImageJ software. All graphical representations of data and statistical analysis were performed in Graphpad Prism (non-parametric Mann–Whitney *U*-test). Error bars represent standard deviation. *n* and *P* values are indicated in figure legends. Based on variance in our data, we have determined the appropriate *n* values via power analysis for live imaging to be a minimum of 7-10 GSCs imaged through two rounds of mitosis. Fixed, end-point analyses were based on previous analyses in the field (Fairchild et al., 2016; Li et al., 2014). Figures were generated using BioRender.com and Adobe Photoshop.

## Supplementary Material



10.1242/develop.202542_sup1 Supplementary information
