# Supplementary material for: Mating-induced Ecdysone in the testis disrupts soma-germline contacts and stem cell cytokinesis
Source: Development. 2024 Jun 4;151(11):dev202542. doi: 10.1242/dev.202542 (PMC11190578; doi:10.1242/dev.202542)
Supplement: Supplementary information [file develop-151-202542-s1.pdf]

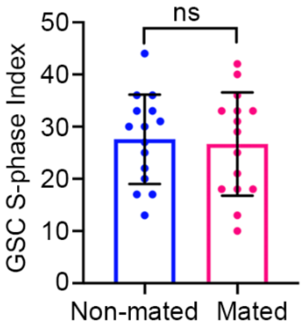


**Fig. S1.** S phase index of GSCs in non-mated vs. mated males. A minimum of 14 testes were analyzed per condition. As visualizing F-actin in germ cells increases the ability to identify GSC-niche interfaces, we believe our results may differ from previous reports due to capturing a fuller complement of the GSC population in our analyses. Error bar: s.d. Mann-Whitney test. n=2 trials.

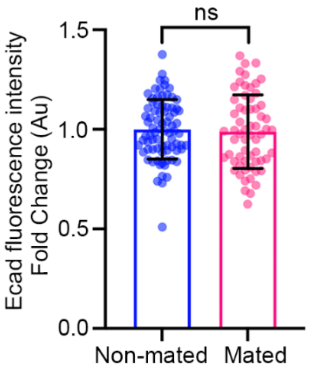


**Fig. S2.** Quantification of E-cad fluorescence intensity at niche-GSC interfaces represented as fold change relative to non-mated controls (n=a minimum of 62 interfaces in at least 11 testes). Error bar: s.d. Mann-Whitney test. n=3 trials.

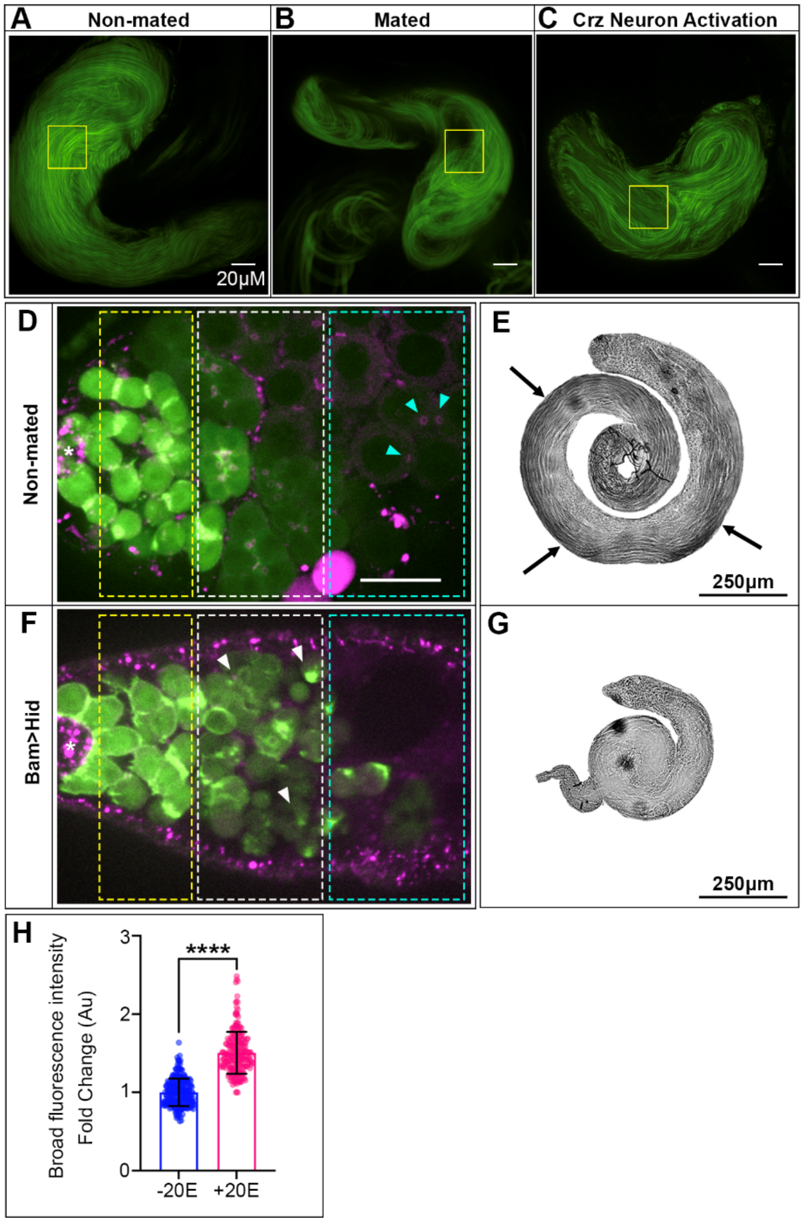


**Fig. S3.** (A-C) 63X fixed images of fluorescently labeled sperm (don juan::GFP) within seminal vesicles from (A) non-mated, (B) mated, and (C) Crz neuron activated males. Yellow boxes highlight the density of sperm across conditions. Scale bar: 20μm. (D,F) Live stills of the apical tip of the testis of (D) non-mated and (F) Bam>Hid testes. Yellow boxes show healthy early germ cells in both; white boxes show intact 4 to 16-cell cysts in (D) and dying cysts in (F) (white arrow heads); cyan boxes show differentiating spermatocytes indicated by ring canals (cyan arrow heads) in (D) and absence of spermatocytes in (F). Scale bar: 20μm (E,G) 10X brightfield images of testes from (E) non-mated males containing sperm (arrows) and from (G) Bam>Hid testes lacking sperm and significantly reduced in overall size. Scale bar: 250μm. (H) Quantification of broad fluorescence intensity represented as fold change in testis soma (n≥230 somatic nuclei in 3 testes). Error bar: s.d. Mann-Whitney test. n=1 trial.

Development • Supplementary information

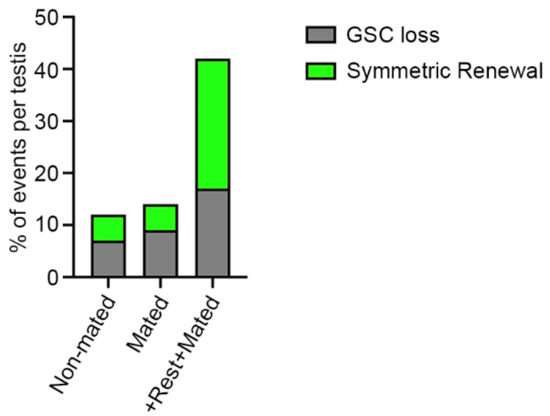


**Fig. S4.** Number of events for GSC loss and symmetric renewal in testes from non-mated, mated, and +Rest+Mated males. A minimum of 11 testes were analyzed for quantification of GSC loss and symmetric renewal events. n≥2 trials.
