# Supplementary material for: Mating-induced Ecdysone in the testis disrupts soma-germline contacts and stem cell cytokinesis
Source: Development. 2024 Jun 4;151(11):dev202542. doi: 10.1242/dev.202542 (PMC11190578; doi:10.1242/dev.202542)
Supplement: Supplementary file 1 [file develop-151-202542_review_history.pdf]

Mating-induced ecdysone in the testis disrupts soma-germline contacts and stem cell cytokinesis

Tiffany V Roach and Kari F Lenhart
DOI: 10.1242/dev.202542

Editor: Cassandra Extavour

Review timeline
Original submission: 13 November 2023
Editorial decision: 12 January 2024
First revision received: 5 April 2024
Accepted: 29 April 2024

Original submission

First decision letter

MS ID#: DEVELOP/2023/202542

MS TITLE: Mating-induced ecdysone in the testis disrupts soma-germline contacts and stem cell cytokinesis

AUTHORS: Tiffany V Roach and Kari F Lenhart

Please accept my apologies for the delay in getting a decision to you on this manuscript. The holiday period seems to have caused some delay in receiving reviewer reports, but the last one finally arrived a few days ago. I have therefore now received all the referees reports on the above manuscript, and have reached a decision. The referees' comments are appended below, or you can access them online: please go to BenchPressand click on the 'Manuscripts with Decisions' queue in the Author Area. As you can see, all reviewers are very enthusiastic about the work, and I agree with their assessment. Congratulations on this extremely interesting and well done study, and thank you for submitting it to Development.

We would like to publish a revised manuscript in Development, provided that the referees' comments can be satisfactorily addressed. As you will see, all comments are fairly minor. Please attend to all of the reviewers' comments in your revised manuscript and detail them in your point-by-point response. If you do not agree with any of their criticisms or suggestions explain clearly why this is so. If it would be helpful, you are welcome to contact us to discuss your revision in greater detail. Please send us a point-by-point response indicating your plans for addressing the referees' comments, and we will look over this and provide further guidance.

Reviewer 1

Advance summary and potential significance to field

Roach and Lenhart provide an exciting model for how mating disrupts the contacts between germline and soma in the Drosophila testis, resulting in defects in proper germline stem cell cytokinesis. The work is very relevant and adds to the field of developmental and stem cell biology by cleverly exploring mechanistically how a ‘stress’ phenomenon such as mating contributes to the homeostasis of the male germline stem cell niche. The manuscript is very well written, the data are

© 2024. Published by The Company of Biologists under the terms of the Creative Commons Attribution License (https://creativecommons.org/licenses/by/4.0/).

strong, and the images are beyond convincing and beautiful. The manuscript would be very suitable for publication in this journal upon some minor revisions (see points below).

Comments for the author

1) The authors show via live imaging (Fig 1E) and Edu stains (fixed) that the cell cycle doesn't change upon mating. It would be good if the authors could offer a potential explanation as to why their results differ from data in Ameku and Niwa,2016 and Malpe et al. 2020 (not to say the authors' data are wrong obviously, but since they are diverging from previously reported data it would be interesting to know their interpretation of these results and why they look different).

2) Since EGFR activation is decreased upon mating (dpERK is lower in Fig 2G-I), and germline-secreted Spitz is the main ligand that activates EGFR in the soma are Spitz levels also decreased upon mating? The use of a Spitz-lacZ (BL10462) or a Spitz antibody (AB_528474) could be beneficial in understanding the levels of the ligand as mating could be influencing Spitz expression in the germline therefore affecting EGFR signaling in cyst cells.

3) If Ecdysone/Broad act to counteract Rac via activation of Rho (proposed model in Fig 4), then why is dpERK decreased (Fig 2G-I)? Low dpERK levels suggest that either Ecdysone/Broad act at the receptor level (i.e. repressing EGFR) or in the germline by inhibiting the production of normal Spitz levels. Perhaps if there is evidence showing that lack of proper ensheathment leads to lowered overall dpERK signaling (as perhaps a feedback loop?) the authors could cite to justify their results.

4) The results upon ecdysone feeding are very exciting, and *sufficiency* for increased ecdysone levels triggering abscission failure was demonstrated by the authors in Fig 3. Can the authors show *requirement* for increased ecdysone through perhaps a conditional depletion of ecdysone synthesis (maybe using a drug/temperature inducible system to deplete genes involved in ecdysone synthesis in the whole body) or depletion of the EcR in cyst cells just during mating? The male isolation experiments (Fig4) are helpful in building this argument, but do not exclude the contribution of perhaps another pathway.

-minor comments (perhaps addressed in discussion/future experiments):
1)The optogenetics experiments are very interesting and novel. To support their claims, can the authors show evidence that more ejaculations actually happen upon Crz>CsChrimson activation?

2)Yang and Yamashita 2015 showed that cyst cell/spermatogonial death prevents further loss of GSCs upon starvation. Hence, it is possible that killing spermatogonia with bam>Hid could be sending similar 'survival' or preservation signals for GSCs, which could possibly interfere with abscission. This could be discussed in the paper.

3)The authors come up with a very clever and more reliable assay for cyst cell ensheathment, which will certainly benefit the field. A question arises though – does Ecad expression overall decreases in these cells upon mating, or is it more cytoplasmic instead of present at the intersections? I'm assuming the authors believe Ecad just changes localization upon mating, but at this point they can't exclude a loss of overall Ecad expression. Quantification of the ratio of intersection/cytoplasmic signal would perhaps address this issue.

Reviewer 2

Advance summary and potential significance to field

In this Research Report, Roach and Lenhart describe the mechanism underlying male GSC abscission failure in response to mating. They find that mating leads to reduced GSC-somatic cell adhesion and thus reduced cytokinesis. The authors show that this is mediated by EGFR and ecdysone signaling but not by depletions in differentiated germ cells. They also show that reduction in GSC abscission is dynamic – GSC cytokinesis/abscission failure is responsive to multiple mating bouts. Interestingly, the authors propose that this phenomenon may be a way for the testis to restore the GSC pool. The clearly described data support the authors' conclusions. This manuscript is suitable

© 2024. Published by The Company of Biologists under the terms of the Creative Commons Attribution License (https://creativecommons.org/licenses/by/4.0/).

for publication after the authors have address the major, moderate, and minor concerns delineated below.

Comments for the author

Summary
In this Research Report, Roach and Lenhart describe the mechanism underlying male GSC abscission failure in response to mating. They find that mating leads to reduced GSC-somatic cell adhesion and thus reduced cytokinesis. The authors show that this is mediated by EGFR and ecdysone signaling but not by depletions in differentiated germ cells. They also show that reduction in GSC abscission is dynamic – GSC cytokinesis/abscission failure is responsive to multiple mating bouts. Interestingly, the authors propose that this phenomenon may be a way for the testis to restore the GSC pool. The clearly described data support the authors' conclusions. This manuscript is suitable for publication after the authors have address the major, moderate, and minor concerns delineated below.

Major Concerns
Lines 105-114: To say that mating specifically disrupts soma-germline interactions at the encystment step, the authors need to also show that GSC-hub adhesion is not affected by mating. In addition, presumably, the cyst cells are still present but it would be nice to show that they are there.

Moderate Concerns
Lines 116-118: These two intracellular pathways are mentioned, but only Erk activation is assessed. It would be nice to determine if Vav activation and/or cytoskeletal organization is altered in mated males compared to non-mated. Alternatively, this paragraph can be reorganized slightly to focus on Erk first and then mention the possibility of Vav and the cytoskeleton contributing. Additionally, the authors can strengthen the discussion by speculating on which targets of Erk may be involved in GSC abscission.
Line 138: Evidence of sperm depletion via optogentic activation of Crz neurons should be shown either in the main figure or as a supplemental figure.

Lines 136-139 and Figure 3A-B'' and D: To improve the flow of logic, the first part of this figure can be rearranged such that panel A shows the experimental paradigm (currently B'), panel B' shows the example of abscission failure (currently A), panel B'' stays the same, showing normal GSC abscission. The main text can be modified to match the reordered panels

Line 143 and Figure 3C: Instead of, or to complement, the C' schematic, it would be nice to see an IF image of the testis from Bam > Hid

Lines 173-175: Can the authors show that 20E feeding leads to the expected increase in Broad expression? Or perhaps include a reference that has shown this previously?

Line 190: Since GSC abscission dynamics seem to be a part of normal testis mating physiology, perhaps use "response" instead of "defects"

Minor Concerns
Lines 29-30: would be nice to see at least one example of a mechanism used to balance self-renewal and differentiation

Line 109: "F.2C" should be Fig. 2C (in fact, throughout the text, there is some variability in figure notations)

Lines 110-112: for E-cad quantification soma-germline interface, it would be nice to see an outline of the region that was measured, perhaps with a dotted outline or some other way to highlight, in a third column if necessary (Fig. 2B-E)

Lines 128-147: no mention of Figure 3A…

Line 138: Perhaps also add the reference for the work that identified Crz neuron activation mediates sperm depletion in the absence of mating would be Thayer et al 2012 PNAS

© 2024. Published by The Company of Biologists under the terms of the Creative Commons Attribution License (https://creativecommons.org/licenses/by/4.0/).

(DOI:10.1073/pnas.1218246109).

Line 140: referring to “more severe depletion” - add in a sentence that indicates with the average sperm depletion level is when Crz neurons are activated so that readers can compare to the complete germ cell depletion observed when Hid is expressed

Line 175: The figure notation should also include H

Line 190: in the figure notation, add “compare A to B”

Line 237: Since abscission failure happens in response to a normal biological process, mating, this reviewer finds the speculation that this is an adaptive function to be quite compelling. To expand the discussion, the authors may want to allude to future studies that could address this speculation

Line 484: add in “(green)” after “ABD-moe::GFP and “(magenta)” after “Myo-mCherry

For all quantifications: Please indicate what individual data points refer to in the graphs (i.e., each testis analyzed, somatic nuclei, etc)

Reviewer 3

Advance summary and potential significance to field

Roach and Lenhart extend on the Lenhart lab’s very interesting line of work studying germ cell abscission in the D. melanogaster testis. The Lenhart lab previously showed that germline stem cell (GSC) abscission is delayed in old males, yielding GSCs that remain attached to both the niche and a short chain of daughter cells, which are normally released into the testis to undergo differentiation. This abscission defect increases with age, but whether the mating status of the male has any effect was unknown. Linking organismal behavior to stem cell activity is a challenge that the authors admirably and effectively address in their current manuscript. By combining elegant genetic strategies with live imaging, they show that mating increases ecdysone signaling in the somatic cells of the testis. Interestingly, feeding ecdysone is sufficient to cause the abscission defect in the absence of mating. The abscission defect is reversible, and is accompanied by weakened soma-germline contacts, which are also decreased after ecdysone feeding alone. Together these data represent a significant advance in the field, given that little is known about the effects of organismal behavior on stem cell function in general. The manuscript is very clearly written, the figures contain high quality data presented in a very accessible manner, and the results provide strong support for the conclusions.

Comments for the author

The following minor changes are suggested for the text:

Results section

1) Line 73: “This results in a chain of connected daughter cells remaining attached to the niche (Fig.1C), preventing release of differentiating germ cells.”

Please explain how one might distinguish between a prevention of germ cell release versus a delay in the process. It may help to mention that the authors have previously showed that preventing abscission decreases the number of differentiating germ cells.

2) Line 103: “we established a method for detecting subtle disruptions in soma-germline contacts focusing on 2-cell cysts, as encystment is complete by this stage (Fig. 2A).” There are nice data in Figure 2 regarding a new way to approximate encystment defects at a very early stage of germ cell differentiation (two cell stage) using E-cadherin immunolocalization. Flies expressing dominant-negative Rac are controls. A “subtle” defect in encystment is mentioned - perhaps say a bit more about what this means. For instance, is the somatic permeability barrier that develops at the four-

© 2024. Published by The Company of Biologists under the terms of the Creative Commons Attribution License (https://creativecommons.org/licenses/by/4.0/).

cell stage also disrupted in dominant-negative Rac flies? If permeability has been assayed before in this genotype please mention it here, and if not, state this caveat.

3) Regarding the clever use of optogenetic activation of ejaculation, on Line 387: "…We visually confirmed successful activation of neurons and resulting ejaculation with this paradigm." Kindly mention the penetrance of this phenotype in the text. Did it occur in some or all flies? Were flies that passed the visible test used for further analysis? Please move this information from the materials and methods to the results section to help readers can evaluate the results appropriately.

4) Regarding germ cell ablation via ectopic bam expression on Line 141: "By expressing an apoptotic factor Hid, in differentiating germ cells via Bam-Gal4, we can retain the niche and resident stem cells, but effectively kill all germ cells past the 2-cell cyst stage (Fig.3C')." Please mention in the text how the effectiveness of germ cell depletion in the BamGal4; UAS-hid males is assessed.

Discussion section

5) this section is very well written, combining and building on several prior observations in the field in an interesting new way. A minor suggestion: in the Abstract (Line 24) the authors state "….Together, these results suggest a model whereby acute mating results in hormonal changes that negatively impact GSC cytokinesis but preserves the stem cell population." What is the evidence that the "preserved" stem cells (presumably, GSCs with abscission failure) are functional? The live imaging is lovely but has limitations as well. Could GSCs with abscission failure eventually detach and die, and become replaced by other germ cells over time? Perhaps expand on this point in the Discussion.

Methods section
6) In the methods, you could insert full genotypes so that readers do not have to log in to an organismal database to interpret the work.

Example, Line 280: "UAS CsChrimson (Gift from Lisa Shao; BDSC: 55136)"

Instead: UAS CsChrimson (Gift from Lisa Shao; BDSC: 55136; w[1118];P{y[+t7.7] w[mc]=20XUAS-IVS-CSChrimson.mVenus}attP2)

7) Line 396: "Young virgin males" Please specify the age of the males used here.

Figures section

8) The figures are wonderfully prepared, with high quality images and very clear labeling that make them straightforward to interpret throughout the manuscript. One very minor exception is found in panels 4F and H.
Can the authors shift the black boxes in the leftmost portions of panels F and H to the other CySC? (e.g., move boxes from the right to left CySCs). It would be great if the enlarged views in the main panels matched the smaller diagrams.

First revision

Author response to reviewers' comments

We have carefully considered each of the comments from all three reviewers and believe we have addressed each of their specific concerns regarding the manuscript.

Reviewer 1 Comments for the Author:

© 2024. Published by The Company of Biologists under the terms of the Creative Commons Attribution License (https://creativecommons.org/licenses/by/4.0/).

1) The authors show via live imaging (Fig 1E) and Edu stains (fixed) that the cell cycle doesn't change upon mating. It would be good if the authors could offer a potential explanation as to why their results differ from data in Ameku and Niwa, 2016 and Malpe et al. 2020 (not to say the authors' data are wrong, obviously, but since they are diverging from previously reported data it would be interesting to know their interpretation of these results and why they look different).
We agree there is a discrepancy between our directly calculated cycling rates and changes in mitotic index previously reported under mating conditions. We believe a few factors might be at play.

1. In previous work, GSCs were identified via labeling with Vasa, a germ cell- specific cytoplasmic marker, to count germ cells adjacent to fasciclin III stained hubs. By contrast, all of our analyses were conducted with the nos- ABDmoe::GFP marker to label F-actin in the germline. This technique permits us to directly visualize the F-actin enrichments at GSC-hub interfaces, and consistently leads us to identify a greater number of GSCs than is typically reported (Lenhart and DiNardo, 2015; Lenhart et al., 2019), as we can confidently identify GSCs positioned above and below the hub, not just those surrounding it. As we know that there is significant variability in GSC cycling rates (Lenhart and DiNardo), it is possible that our analyses permitted us to identify a fuller complement of the GSC population which accounts for the discrepancy with previous reports.
2. The total n used in previous fixed analyses to determine mitotic index were exceptionally high (>500 testes). It is possible that our mating paradigm induces a subtle increase in cycling rates only detectable with substantial sampling. However, even if this is the case, any change requiring a dramatic increase in sample size would not be a large enough deviation from typical GSC cycling rates for us to interpret the phenotype as significantly contributing to abscission failure.

2) Since EGFR activation is decreased upon mating (dpERK is lower in Fig 2G-I), and germline-secreted Spitz is the main ligand that activates EGFR in the soma, are Spitz levels also decreased upon mating? The use of a Spitz-lacZ (BL10462) or a Spitz antibody (AB_528474) could be beneficial in understanding the levels of the ligand as mating could be influencing Spitz expression in the germline, therefore affecting EGFR signaling in cyst cells.
In our hands, spitz-LacZ showed no distinguishable localization to quantify. We also attempted to quantify EGF receptor abundance to confirm if the effects we observe are due to changes at the receptor level, but the EGFR antibody localized exclusively to the muscle sheath. Previous word identified a genetic interaction between Ecdysone and EGFR leading to decreased EGFR activity (Qian et al., 2014); however, the mechanism underlying that interaction was not explored. In our future work, we intend to more deeply explore the connection between ecdysone and EGFR.

3) If Ecdysone/Broad act to counteract Rac via activation of Rho (proposed model in Fig 4), then why is dpERK decreased (Fig 2G-I)? Low dpERK levels suggest that either Ecdysone/Broad act at the receptor level (i.e. repressing EGFR) or in the germline by inhibiting the production of normal Spitz levels. Perhaps if there is evidence showing that lack of proper ensheathment leads to lowered overall dpERK signaling (as perhaps a feedback loop?) the authors could cite to justify their results. The reviewer is correct that multiple inputs might be at play to alter dpErk activity and/or somatic encystment of the germline. As there is no existing evidence that lack of proper ensheatment leads to lower dpERK activity and as previous genetic analyses indicate ecdysone acts antagonistically to EGF signaling (Qian et al., 2014), we have simplified both our discussion in the text and our model figure to focus more specifically on a potential role for Ecdysone/Broad in repressing EGFR activity in an as yet unspecified manner.

4) The results upon ecdysone feeding are very exciting, and *sufficiency* for increased ecdysone levels triggering abscission failure was demonstrated by the authors in Fig 3. Can the authors show *requirement* for increased ecdysone through perhaps a conditional depletion of ecdysone synthesis (maybe using a drug/temperature inducible system to deplete genes involved in ecdysone synthesis in the whole body) or depletion of the EcR in cyst cells just during mating? The male isolation experiments (Fig4) are helpful in building this argument, but do not exclude the contribution of perhaps another pathway.
We thank the reviewer for this important suggestion. We have now conducted experiments expressing a dominant-negative EcR-B2 specifically in somatic cells of testes and combined this with 2 days of mating. Excitingly, we found a significant decline in GSC abscission failure in these

© 2024. Published by The Company of Biologists under the terms of the Creative Commons Attribution License (https://creativecommons.org/licenses/by/4.0/).

conditions, suggesting that Ecdysone signaling is both necessary and sufficient to induce defects in stem cell cytokinesis. This data has been incorporated in Figure 3 of the manuscript and is discussed in the revised results section.

-minor comments (perhaps addressed in discussion/future experiments):

1) The optogenetics experiments are very interesting and novel. To support their claims, can the authors show evidence that more ejaculations actually happen upon Crz>CsChrimson activation?

When Crz>CsChrimson males are exposed to red light, 100% of the population immediately show behaviors of ejaculation, which we observed using a standard dissection scope. Exposure to red light did not induce this behavior in any sibling controls. This information has been added to the materials and methods section. Additionally, we analyzed sperm density within seminal vesicles (using don juan::GFP) and found optogenetic activation of Crz neurons leads to a decrease in the amount of sperm similar to the decrease observed upon 2 days of mating. This data has been incorporated into Fig.S3.

2) Yang and Yamashita 2015 showed that cyst cell/spermatogonial death prevents further loss of GSCs upon starvation. Hence, it is possible that killing spermatogonia with bam>Hid could be sending similar 'survival' or preservation signals for GSCs, which could possibly interfere with abscission. This could be discussed in the paper.

The idea that there might be a survival or preservation signal feeding back to GSCs under catastrophic loss of differentiating germ cells is an exciting hypothesis currently being investigated in our lab. However, in this work, we show that catastrophic loss of differentiated germ cells has no effect on the completion of GSC abscission. This result, combined with evidence of ecdysone being both sufficient and necessary for causing GSC abscission failure (Fig.3F-I), suggests that mating-induced abscission failure is not due to loss of germ cells, but rather the increased ecdysone signaling.

3) The authors come up with a very clever and more reliable assay for cyst cell ensheathment, which will certainly benefit the field. A question arises though - does Ecad expression overall decreases in these cells upon mating, or is it more cytoplasmic instead of present at the intersections? I'm assuming the authors believe Ecad just changes localization upon mating, but at this point they can't exclude a loss of overall Ecad expression. Quantification of the ratio of intersection/cytoplasmic signal would perhaps address this issue.

This is a critical point that we have now addressed. GSCs associate with the niche through adherens junctions highly enriched for E-cadherin. Thus, we quantified Ecad at hub-GSC interfaces in the same testes in which we previously quantified Ecad at soma- germline contacts of 2-cell cysts. Importantly, we found no difference in Ecad levels at GSC-niche interfaces from non-mated vs mated testes (Fig.S2), suggesting that there is not a global decrease in Ecad levels. Although it would be interesting to know whether Ecad is downregulated specifically in soma in response to mating, the proposed approach of taking membrane to cytoplasmic ratios of Ecad in somatic cells is not technically feasible. Given the abnormal morphology of somatic cells in the testis, it is not possible to create Z-projections (even with mosaic labeling of soma) to quantify full membrane vs. cytoplasmic Ecad. Quantification of cytoplasmic Ecad can also not be accurately achieved from a single Z plane (as was done with soma-germline contacts) because the somatic cells are exceptionally thin with little cytoplasm visible in a single plane.

Reviewer 2 Comments for the Author:

Major Concerns

Lines 105-114: To say that mating specifically disrupts soma-germline interactions at the encystment step, the authors need to also show that GSC-hub adhesion is not affected by mating. In addition, presumably, the cyst cells are still present but it would be nice to show that they are there.

We have now quantified Ecad at hub-GSC interfaces in the same testes in which we previously quantified Ecad at soma-germline contacts of 2-cell cysts. We found no difference in Ecad levels at GSC-niche interfaces from non-mated vs mated testes (Fig.S2), suggesting that GSC-niche adhesion is intact. Moreover, Tj staining of somatic nuclei in Fig.2G-H and Fig.3C-D indicates that cyst cells are present within the testis.

© 2024. Published by The Company of Biologists under the terms of the Creative Commons Attribution License (https://creativecommons.org/licenses/by/4.0/).

Moderate Concerns

Lines 116-118: These two intracellular pathways are mentioned, but only Erk activation is assessed. It would be nice to determine if Vav activation and/or cytoskeletal organization is altered in mated males compared to non-mated. Alternatively, this paragraph can be reorganized slightly to focus on Erk first and then mention the possibility of Vav and the cytoskeleton contributing. Additionally, the authors can strengthen the discussion by speculating on which targets of Erk may be involved in GSC abscission.
It is well-established that somatic encystment downstream of EGFR is mediated by cytoskeletal changes (Rac) rather than transcriptional targets downstream of Erk (Sarkar et al., 2007; Kiger et al., 2000). Additional studies confirmed that changes in cytoskeletal regulation (disruption of profilin activity) are sufficient to disrupt somatic encystment (Shields et al., 2014). However, given that both Rac and Erk activation lie downstream of EGFR (and in the absence of reliable Vav activity markers) we use dpERK intensity as a proxy for EGFR activity which will impact both arms of the pathway. The approach of using dpERK intensity as a read-out for potential encystment defects has been used previously in our field by a number of labs (Gupta et al, 2018; Fairchild et al, 2016). We have clarified this point in the results/discussion.
Line 138: Evidence of sperm depletion via optogenetic activation of Crz neurons should be shown either in the main figure or as a supplemental figure.
We now show that Crz>CsChrimson males have seminal vesicles that are less densely packed with sperm as labeled by don juan::GFP similar to those from mated males compared to non-mated males (Fig.S3A-C).

Lines 136-139 and Figure 3A-B’’ and D: To improve the flow of logic, the first part of this figure can be rearranged such that panel A shows the experimental paradigm (currently B’), panel B’ shows the example of abscission failure (currently A), panel B’’ stays the same, showing normal GSC abscission. The main text can be modified to match the reordered panels
As per your fourth minor concern, Fig.3A was not referenced in the text and is redundant to include here. This figure has been reorganized excluding this panel of images, which should help the logic flow.

Line 143 and Figure 3C: Instead of, or to complement, the C’ schematic, it would be nice to see an IF image of the testis from Bam > Hid
We have added supplemental live stills of control testes from non-mating conditions and testes with Bam>Hid expression (Fig.S3D,F), highlighting the morphological changes used to confirm germ cell depletion in that figure (Fig.S3B)

Lines 173-175: Can the authors show that 20E feeding leads to the expected increase in Broad expression? Or perhaps include a reference that has shown this previously? We have added quantification of Broad fluorescence intensity upon 20E feeding to Fig.S3 and do, indeed, observe a significant increase compared with vehicle-fed controls (n=3 testes, 230 cells) (Fig.S3H)

Line 190: Since GSC abscission dynamics seem to be a part of normal testis mating physiology, perhaps use “response” instead of “defects”
During homeostasis, there is near 100% success in GSC abscission. In addition, we have previously shown that abscission failure correlates with decreased numbers of differentiating germ cells and that simply restoring successful GSC abscission is sufficient to rescue the numbers of differentiating germ cells (Lenhart et al 2019).
Finally, the abscission failure with mating is caused by a non-autonomous change to the somatic population—the soma responds to mating and the germline exhibits defects as a consequence. Thus, while we agree that abscission failure is clearly happening in response to mating, we believe this should still be characterized as a defect given the negative effect it has on the tissue.

Minor Concerns

Lines 29-30: would be nice to see at least one example of a mechanism used to balance self-renewal and differentiation
We have now added a phrase to this effect.

Line 109: “F.2C” should be Fig. 2C (in fact, throughout the text, there is some variability in figure notations)
We have revised this in the text.

© 2024. Published by The Company of Biologists under the terms of the Creative Commons Attribution License (https://creativecommons.org/licenses/by/4.0/).

Lines 110-112: for E-cad quantification soma-germline interface, it would be nice to see an outline of the region that was measured, perhaps with a dotted outline or some other way to highlight, in a third column if necessary (Fig. 2B-E)
We have added a dotted line to the magnified view of the control 2-cell cyst to indicate the soma-germline interface where Ecad fluorescence was quantified (Fig.2B).

Lines 128-147: no mention of Figure 3A...
We have removed this panel of images from the figure.

Line 138: Perhaps also add the reference for the work that identified Crz neuron activation mediates sperm depletion in the absence of mating would be Thayer et al 2012 PNAS (DOI:10.1073/pnas.1218246109).
This reference has now been added.

Line 140: referring to “more severe depletion” – add in a sentence that indicates with the average sperm depletion level is when Crz neurons are activated so that readers can compare to the complete germ cell depletion observed when Hid is expressed
We have added supplemental images to Fig.S3 showing stills from live imaging of non- mated males versus those with expression of Hid under Bam control. Regions of germ cell death (white box) and absence of spermatocytes (cyan box) are indicated in the Bam>Hid images (Fig.S3F). Additionally, we have clarified in the text that Bam>Hid testes completely lack sperm and, due to the absence of all differentiated germ cells, are dramatically smaller than control testes (Fig.S3E,G).

Line 237: Since abscission failure happens in response to a normal biological process, mating, this reviewer finds the speculation that this is an adaptive function to be quite compelling. To expand the discussion, the authors may want to allude to future studies that could address this speculation
We agree this is an interesting point for further study and have added a statement to this effect in the discussion.
Line 484: add in “(green)” after “ABD-moe::GFP and “(magenta)” after “Myo-mCherry For all quantifications: Please indicate what individual data points refer to in the graphs (i.e., each testis analyzed, somatic nuclei, etc)
We have added this information to the figure legends.

Reviewer 3 Comments for the Author:
The following minor changes are suggested for the text:
Results section

1) Line 73: “This results in a chain of connected daughter cells remaining attached to the niche (Fig.1C), preventing release of differentiating germ cells.” Please explain how one might distinguish between a prevention of germ cell release versus a delay in the process. It may help to mention that the authors have previously showed that preventing abscission decreases the number of differentiating germ cells.
We agree that our 24 hour imaging precludes us from determining that *all* abscission failure events prevent release of differentiating daughters. However, as this reviewer notes, our lab has previously found that abscission failure correlates with a decrease in the number of differentiating germ cells and that this can be completely rescued simply by restoring successful GSC abscission in the tissue. Thus, abscission failure does prevent release of differentiating cells from the niche, even if a specific instance of abscission failure may not.

2) Line 103: “we established a method for detecting subtle disruptions in soma-germline contacts focusing on 2-cell cysts, as encystment is complete by this stage (Fig. 2A).” There are nice data in Figure 2 regarding a new way to approximate encystment defects at a very early stage of germ cell differentiation (two cell stage) using E-cadherin immunolocalization. Flies expressing dominant-negative Rac are controls. A “subtle” defect in encystment is mentioned – perhaps say a bit more about what this means. For instance, is the somatic permeability barrier that develops at the four-cell stage also disrupted in dominant-negative Rac flies? If permeability has been assayed before in this genotype please mention it here, and if not, state this caveat.

© 2024. Published by The Company of Biologists under the terms of the Creative Commons Attribution License (https://creativecommons.org/licenses/by/4.0/).

While the somatic permeability barrier has not been assessed with Rac inhibition, there are multiple lines of evidence suggesting that Rac loss-of-function induces a subtle defect in encystment:

1. Severe disruption of encystment causes dramatic changes to testis morphology (Lenhart et al, 2019; Sarkar et al., 2007; Kiger et al., 2000). Somatic expression of DN-Rac1 does not alter global morphology.
2. Rac was originally identified as a genetic modifier in a hypomorphic spitz mutant background. Depletion of Rac on its own in the absence of this sensitized background does not result in germ cell overproliferation (the hallmark of testes with severely disrupted encystment) (Sarkar et al., 2007).
3. Our lab has previously shown that somatic expression of DN-Rac1 is sufficient to induce similar defects in GSC abscission as expression of DN-egfr. While we observed germ cell overproliferation with loss of EGFR activity, we did not observe this phenotype with DN-Rac1 expression- despite the similar effects on GSC cytokinesis.

Taken together, these data strongly suggest that somatic expression of DN-Rac1 subtly disrupts encystment but in a manner sufficient to inhibit GSC abscission. A concise rationale for the description of Rac inhibition as “subtle” has been added to the text of the manuscript.

3) Regarding the clever use of optogenetic activation of ejaculation, on Line 387: “…We visually confirmed successful activation of neurons and resulting ejaculation with this paradigm.” Kindly mention the penetrance of this phenotype in the text. Did it occur in some or all flies? Were flies that passed the visible test used for further analysis? Please move this information from the materials and methods to the results section to help readers can evaluate the results appropriately.
We confirmed that 100% of experimental flies (Crz>csChrimson) induced behaviors associated with ejaculation immediately upon exposure to red light. These behaviors were never observed in Gal4 or UAS only controls. We confirmed that experimental flies exhibited this response to light prior to every experiment. We have added this clarification to the text of the manuscript and materials and methods. Finally, we have confirmed (using don juan::GFP) that optogenetic activation of ejaculation results in a decreased density of sperm within the seminal vesicle similar to what we observe after 2 days of mating. Corresponding images have been added to Fig.S3.

4) Regarding germ cell ablation via ectopic bam expression on Line 141: “By expressing an apoptotic factor, Hid, in differentiating germ cells via Bam-Gal4, we can retain the niche and resident stem cells, but effectively kill all germ cells past the 2-cell cyst stage (Fig.3C’).” Please mention in the text how the effectiveness of germ cell depletion in the BamGal4; UAS-hid males is assessed.
Supplemental images have been added to Fig.S3 to show regions of cell death in 4-cell cysts (the stage where Bam is normally expressed) and resulting absence of spermatocytes (Fig.S3F). All dissected Bam>Hid testes were significantly smaller than controls and lacked sperm (Fig.S3E,G).

Discussion section

5) This section is very well written, combining and building on several prior observations in the field in an interesting new way. A minor suggestion: in the Abstract (Line 24) the authors state “….Together, these results suggest a model whereby acute mating results in hormonal changes that negatively impact GSC cytokinesis but preserves the stem cell population.” What is the evidence that the “preserved” stem cells (presumably, GSCs with abscission failure) are functional? The live imaging is lovely but has limitations as well. Could GSCs with abscission failure eventually detach and die, and become replaced by other germ cells over time? Perhaps expand on this point in the Discussion.
Previous (Lenhart and DiNardo, 2015; Lenhart et al., 2019) and unpublished work from our lab has shown that abscission failure can “resolve” in two ways: (1) with the full 4- cell cluster leaving the niche and (2) with the original intercellular bridge eventually completing abscission, resulting in two cells remaining at the niche and two being released. In the latter case, we have observed the remaining GSC-pair complete abscission and the individual, remaining GSC then undergo a completely normal cytokinesis program in the following cell cycle. Thus, one cycle of failed abscission does not predict future cytokinesis failure. In addition, we have previously found that even upon abscission failure, the GSC attached to the niche retains stem cell identity

© 2024. Published by The Company of Biologists under the terms of the Creative Commons Attribution License (https://creativecommons.org/licenses/by/4.0/).

(behavior/gene expression; Lenhart and DiNardo). This suggests that while abscission failure is detrimental to the tissue (decreased differentiating cells), it is not catastrophic. We believe a short-term decrease in production of differentiating cells (abscission failure) that permits a long-term ability to retain the stem cell pool (de-differentiation) has led to mating-induced encystment defects being selected FOR rather than AGAINST. A concise description of this logic has been included in the discussion.

Methods section

6) In the methods, you could insert full genotypes so that readers do not have to log in to an organismal database to interpret the work.
Example, Line 280: "UAS CsChrimson (Gift from Lisa Shao; BDSC: 55136)" Instead: UAS CsChrimson (Gift from Lisa Shao; BDSC: 55136; w[1118];P{y[+t7.7] w[mc]=20XUAS-IVS-CSChrimson.mVenus}attP2)
We have now added the full genotypes for each line that was used in our experiments.

7) Line 396: "Young virgin males" Please specify the age of the males used here.
We have replaced this phrasing with the exact age range that was utilized (0-3day old males).

Figures section

8) The figures are wonderfully prepared, with high quality images and very clear labeling that make them straightforward to interpret throughout the manuscript. One very minor exception is found in panels 4F and H. Can the authors shift the black boxes in the leftmost portions of panels F and H to the other CySC? (e.g., move boxes from the right to left CySCs). It would be great if the enlarged views in the main panels matched the smaller diagrams.
We have adjusted the diagrams to match this description.

Second decision letter

MS ID#: DEVELOP/2023/202542

MS TITLE: Mating-induced ecdysone in the testis disrupts soma-germline contacts and stem cell cytokinesis

AUTHORS: Tiffany V Roach and Kari F Lenhart

I am happy to tell you that your manuscript has been accepted for publication in Development, pending our standard ethics checks.

Reviewer 1

Advance summary and potential significance to field

The work by Roach and Lenhart addresses the psysiological impact of mating in the phenomenon of somatic control of stem cell cytokinesis, an important contribution to the stem cell biology and gametogenesis fields.

Comments for the author

The authors have satisfactory addressed all issues raised by this reviewer, either by text or with more experiments.

Reviewer 2

© 2024. Published by The Company of Biologists under the terms of the Creative Commons Attribution License (https://creativecommons.org/licenses/by/4.0/).

Advance summary and potential significance to field

see previous review report

Comments for the author

The authors have sufficiently addressed major and minor concerns and the manuscript is now suitable for acceptance.

© 2024. Published by The Company of Biologists under the terms of the Creative Commons Attribution License (https://creativecommons.org/licenses/by/4.0/).
